# *Taraxacum officinale* L. in leukemia and lymphoma: current knowledge and prospects for horticulture

**DOI:** 10.3389/fpls.2025.1740142

**Published:** 2026-01-07

**Authors:** Massimiliano Renna

**Affiliations:** Department of Soil, Plant and Food Science – University of Bari Aldo Moro, Bari, Italy

**Keywords:** biofortification, dandelion, elicitation, hydroponic systems, LED spectra, secondary metabolites

## Abstract

Dandelion (*Taraxacum officinale* L.) is a globally distributed medicinal plant rich in phenolic acids, flavonoids, sesquiterpene lactones and pentacyclic triterpenoids. Preclinical studies indicate that dandelion extracts and isolated constituents exert selective cytotoxic and pro-apoptotic effects against various hematological malignancies, including leukemia and lymphoma, via mechanisms such as ROS generation, mitochondrial apoptosis, cell-cycle arrest and inhibition of oncogenic signaling (e.g., PI3K/AKT, STAT3). This mini-review synthesizes current *in vitro* and *in vivo* evidence on the antileukemic and antilymphoma potential of *T. officinale*, emphasizing the phytochemical classes most consistently implicated (notably triterpenoids like taraxasterol and pentacyclic acids) and highlighting methodological limitations of existing studies—dose relevance, lack of pharmacokinetic data and sparse safety profiling. Building on these pharmacological insights, horticultural strategies that can modulate bioactive profiles—controlled environment agriculture, hydroponics, elicitation, light spectral management and targeted nutrient fortification— are evaluated and a practical cultivation-to-clinic roadmap to produce standardized, high-value plant material suitable for translational research is proposed. Critical translational barriers, including standardization of extracts, potential interactions with anticancer drugs (notably tyrosine kinase inhibitors), and the need for rigorous toxicity and human pharmacology studies are also discussed. Finally, prioritized experimental and horticultural studies that would accelerate evidence-based development of dandelion-derived therapeutics for hematologic oncology are outlined, while cautioning against premature clinical use without controlled trials.

## 1 Introduction

Hematological malignancies encompass a diverse array of blood and lymphoid cancers, including acute myeloid leukemia (AML), acute lymphoblastic leukemia (ALL), chronic myeloid leukemia (CML), chronic lymphocytic leukemia (CLL), Hodgkin lymphoma (HL), and non-Hodgkin lymphoma (NHL). These disorders are characterized by uncontrolled proliferation, impaired differentiation of hematopoietic cells, and infiltration of blood-forming tissues (Siegel et al., 2024). Despite advancements in chemotherapeutics, targeted small molecules (e.g., tyrosine kinase inhibitors, BCL-2 inhibitors), and monoclonal antibodies, resistance development, off-target toxicities, and relapse rates remain substantial challenges, particularly in refractory and relapsed cases (Ross, 2000; Oliva et al., 2018; Lin et al., 2019).

The investigation of plant-derived bioactive compounds has accelerated in recent years as researchers seek safe, cost-effective adjuncts or alternatives capable of modulating multiple oncogenic pathways (Dehelean et al., 2021). *Taraxacum officinale* L., commonly known as dandelion, is a widespread perennial herb traditionally employed in folk medicine for its diuretic, hepatoprotective, anti-inflammatory, and digestive properties (Schütz et al., 2006; Martinez et al., 2015). Phytochemical analyses using high-performance liquid chromatography have characterized over 50 compounds in various plant parts—roots, leaves, flowers—classified into sesquiterpene lactones, triterpenes, phenolic acids, flavonoids, and other minor constituents such as polysaccharides and sterols (Schütz et al., 2006; Jedrejek et al., 2019; Fan et al., 2023).

Recent years have seen a burgeoning of preclinical studies assessing the anti-proliferative and apoptotic effects of dandelion extracts on leukemia and lymphoma cell lines. Notably, Ovadje et al. (2012) first reported that dandelion root extract (DRE) selectively induces apoptosis in chronic myelomonocytic leukemia (CMML) cell lines through both extrinsic (caspase-8 activation) and intrinsic (mitochondrial depolarization, cytochrome c release) pathways, while sparing normal peripheral blood mononuclear cells (PBMCs). Following this (Al-Eisawi et al., 2022), demonstrated that solvent-partitioned fractions, particularly the chloroform fraction, exhibit enhanced cytotoxicity against K562 (CML) cells via ROS-mediated DNA damage and caspase-3 activation.

However, translating these findings into clinical applications requires a comprehensive understanding of both the pharmaceutical properties and cultivation factors influencing bioactive compound synthesis.

This narrative review aims to provide an overview of current knowledge regarding *T. officinale* in leukemia and lymphoma, integrating perspectives from horticulture and medicine to identify opportunities for future research and clinical development.

## 2 Botanical characteristics and bioactive compounds

*T. officinale* is a perennial herb characterized by a rosette of deeply lobed leaves and distinctive golden-yellow flowers that bloom throughout much of the year (Verma et al., 2023). The plant exhibits remarkable adaptability, thriving in temperate zones of the Northern hemisphere and demonstrating tolerance to various soil conditions and climatic variations (Fan et al., 2023). This cosmopolitan distribution reflects the species’ robust growth characteristics and ecological plasticity, making it an accessible resource for both traditional medicine and modern pharmaceutical applications.

The dandelion plant consists of several distinct anatomical parts—roots, leaves, and flowers—each contributing unique phytochemical profiles (Tanasa (Acretei) et al., 2025). The root system, which can penetrate deeply into soil substrates, serves as a storage organ for numerous bioactive compounds, particularly triterpenoids and sesquiterpenoid lactones. The aerial parts, including leaves and flowers, are rich in flavonoids, phenolic acids, and other secondary metabolites with demonstrated biological activities (Fan et al., 2023).

**Figure 1:**
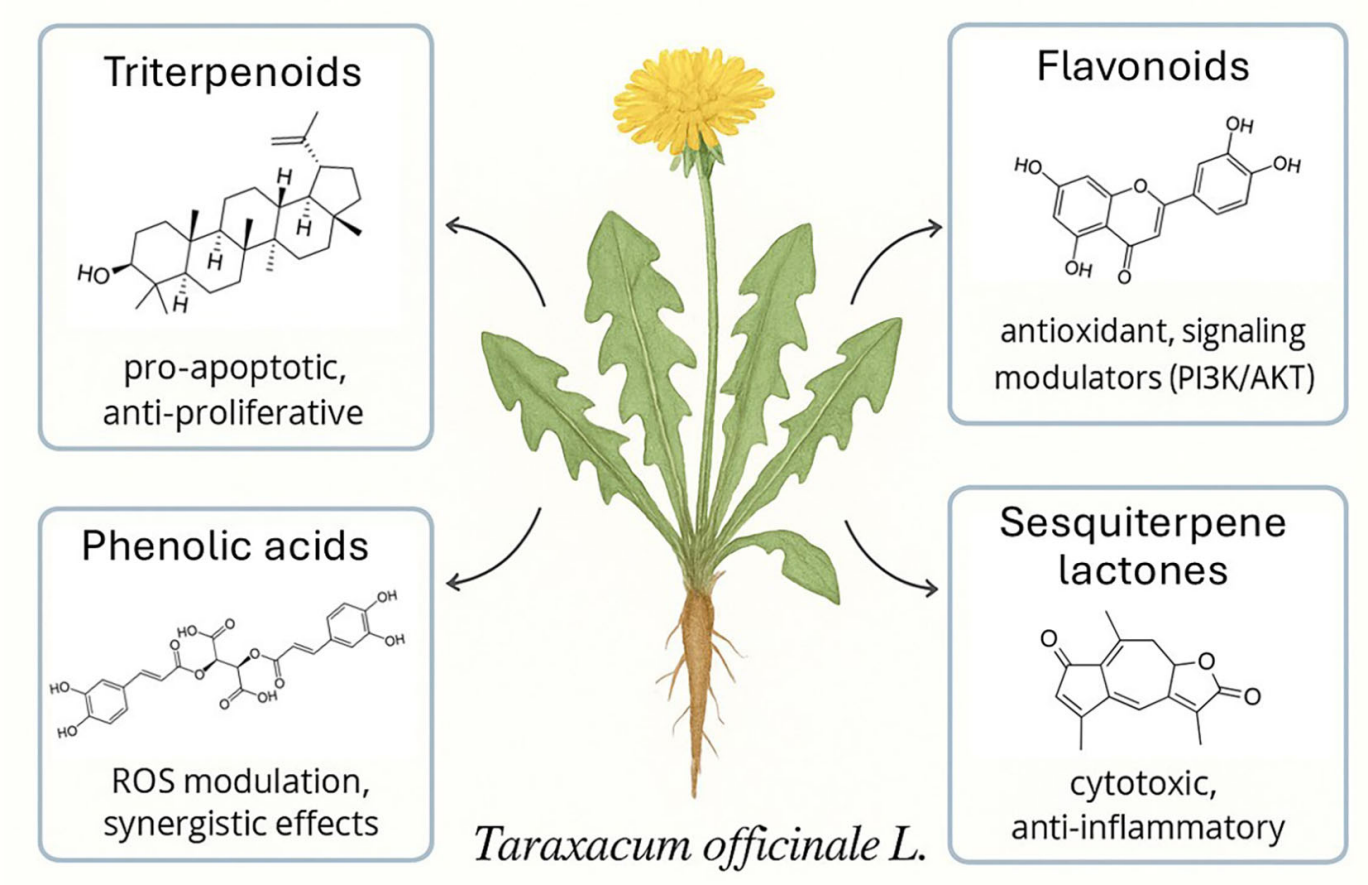
Major classes of bioactive secondary metabolites identified in *Taraxacum officinale* L. and their reported anticancer activities in leukemia and lymphoma models. Representative skeletal structures are shown for each class. Summary of activities: triterpenoids — pro-apoptotic and anti-proliferative; flavonoids — antioxidant and signaling modulators (e.g., PI3K/AKT); phenolic acids — ROS modulation and synergistic/synergistic effects; sesquiterpene lactones — cytotoxic and anti-inflammatory. Horticultural and translational strategies to obtain standardized material for preclinical evaluation are discussed in the main text.

*T. officinale* exhibits a remarkably diverse phytochemical profile, with bioactive compounds distributed across multiple chemical classes (**Figure 1**).

The phenolic acid fraction represents one of the most therapeutically relevant groups, with chicoric acid emerging as a predominant constituent (Schütz et al., 2005; Ivanov, 2015) at concentrations ranging from 8,976 to 52,500 mg/mL depending on extraction method and plant part analyzed (Fulga et al., 2023). Chlorogenic acid, another major phenolic compound, has been quantified at 114.4 mg/mL in 80% ethanolic extracts, while caftaric and caffeic acids show substantial variability (70.4–8,460 mg/mL and 316.8–1,746 mg/mL, respectively) based on tissue type and extraction conditions (Fulga et al., 2023).

Sesquiterpene lactones constitute the largest group of anti-inflammatory and antitumor agents in dandelion, with taraxacin and taraxacerin being predominant (Dhyani et al., 2022; Verma et al., 2023). These compounds possess α-methylene-γ-lactone moieties capable of alkylating nucleophilic residues in cysteine proteases and redox-regulating enzymes, leading to oxidative stress and apoptosis. Characteristic of the Asteraceae family, taraxacin and taraxacerin have demonstrated a key roles in the plant’s hepatoprotective and anticancer activities (Verma et al., 2023).

The dandelion juice contains a rich mixture of flavones and flavonols, predominantly luteolin, quercetin glycosides and apigenin derivates (Schütz et al., 2005; Miłek et al., 2019). The flavonoid content, dominated by luteolin derivatives, ranges from 0.385 to 0.535 mg/mL in leaf and flower extracts (Miłek et al., 2019). Flavonoid constituents, including luteolin and its derivatives, contribute to the plant’s antioxidant capacity and cellular protective effects (Hu et al., 2025). These compounds work synergistically with other phytochemicals to enhance overall therapeutic efficacy, demonstrating the value of whole plant extracts over isolated compounds in many applications.

Triterpenoids represent another important class of bioactive compounds in *T. officinale*, with taraxasterol, β-amyrin, lupeol and taraxarol represent key bioactive components responsible for anticancer activities (Martínez et al., 2023). Taraxasterol, in particular, has attracted attention for its diverse pharmacological properties, including anti-inflammatory, antioxidant, and anticarcinogenic activities (Jiao et al., 2022)

Advanced analytical techniques have proven essential for comprehensive phytochemical characterization. High-performance liquid chromatography with diode array detection (HPLC-DAD) and mass spectrometry (LC-MS, GC-MS) enable precise identification and quantification of individual compounds, while nuclear magnetic resonance (¹H NMR) spectroscopy provides valuable insights into carbohydrate and amino acid profiles (Ambroselli et al., 2024).

Notably, phytochemical concentrations vary significantly according to plant part (root versus aerial tissues), developmental stage, extraction solvent, and growing conditions (Ambroselli et al., 2024). This variability underscores the importance of standardized cultivation and extraction protocols for pharmaceutical applications. The application of multiple complementary analytical methods provides the most comprehensive characterization, essential for quality control and ensuring consistent therapeutic efficacy in leukemia and lymphoma treatments (Alzoman et al., 2019).

## 3 Anticancer mechanisms and synergistic effects with conventional chemotherapy

*T. officinale* exhibits selective anticancer activity against leukemia and lymphoma cells, primarily via apoptosis induction, cell cycle arrest, and modulation of key oncogenic pathways (Ovadje et al., 2012; Chen et al., 2020; Liu et al., 2025).

Dandelion root extract (DRE) triggers caspase-8 activation in chronic myelomonocytic leukemia (CMML) cells, causing mitochondrial destabilization and cell death without harming normal peripheral blood mononuclear cells (Ovadje et al., 2012). In acute myeloid leukemia (AML) models, dandelion extracts act on treatment-naive and chemotherapy-resistant cells through DNA fragmentation, ROS generation, and regulation of pro- and anti-apoptotic proteins, upregulating Bax and p53 while downregulating Bcl-2 (Ahmadi et al., 2023). Notably, dandelion targets cancer stem cells, inhibiting proliferation in both 2D and 3D culture systems. The triterpenoid bauerane induces S-phase arrest and apoptosis in A549 cells via PI3K/AKT and STAT3 inhibition, showing no toxicity to normal cells (Chen et al., 2020).

In lymphoma models, including Hodgkin and non-Hodgkin lymphomas, leaf and root extracts suppress cell viability, with efficacy correlating to hydroxycinnamic acids content (Fulga et al., 2022). Isochlorogenic acid A (ICGA-A) enhances immunotherapy responses by modulating the tumor immune microenvironment and suppressing FAK/PI3K/AKT/mTOR signaling (Wang et al., 2025). Overall, *T. officinale* exerts anticancer effects through intrinsic and extrinsic apoptotic pathways, cell cycle regulation at S and G2/M phases, autophagy activation, oxidative stress modulation, and inhibition of multiple oncogenic signaling cascades, including PI3K/AKT, STAT3, NF-κB, and MAPK, reflecting its multi-target, broad-spectrum potential (Wang et al., 2025).

A key consideration for developing *T. officinale* as a therapeutic agent is its potential to enhance chemotherapy efficacy while reducing toxicity (Nguyen et al., 2019). Preclinical studies show that dandelion extracts, when combined with chemotherapeutic agents such as taxol and mitoxantrone, increase apoptosis and reduce tumor burden in prostate cancer models (Nguyen et al., 2019). Its hepatoprotective properties protect against chemotherapy-induced liver damage by reducing oxidative stress, enhancing antioxidant enzymes, and preventing DNA fragmentation, potentially allowing more intensive treatment or avoiding dose reductions (Elhady and Hassan, 2014; Hfaiedh et al., 2016). Dandelion extracts generally do not interfere with chemotherapy and may exhibit synergistic or additive effects, supporting their use as an adjuvant therapy in hematological malignancies (Korbášová et al., 2022). However, pharmacokinetic interactions with drugs like tyrosine kinase inhibitors (dasatinib, imatinib, nilotinib) must be carefully evaluated, as they could alter drug levels, efficacy, and toxicity (Alzoman et al., 2019). Comprehensive pharmacokinetic and pharmacodynamic studies are essential before clinical combination therapies.

Furthermore, despite extensive preclinical evidence, clinical data on *T. officinale* in hematological malignancies remains limited. The most notable case report describes a 76-year-old male with chronic myelomonocytic leukemia (CMML) whose hematological parameters remained stable while taking papaya leaf extract and dandelion root extract (Rahmat and Damon, 2018). Remarkably, bone marrow blast counts improved substantially during the treatment period, suggesting potential clinical benefit. However, this single case report, while intriguing, provides insufficient evidence for definitive conclusions about clinical efficacy.

The paucity of clinical trials represents a significant knowledge gap that must be addressed to advance dandelion from laboratory findings to clinical practice. Well-designed clinical studies are needed to establish appropriate dosing regimens, assess safety in cancer patient populations receiving concurrent therapies, and definitively evaluate therapeutic efficacy (Cord et al., 2025).

However, several considerations are important for therapeutic applications. First, allergic reactions are possible, particularly in individuals with known sensitivity to plants in the Asteraceae family (Tanasa (Acretei) et al., 2025). Second, the potential for drug-herb interactions, though not extensively studied, warrants caution when combining dandelion with medications metabolized by cytochrome P450 enzymes or transported by specific drug transporters.

## 4 Cultivation and elicitation techniques for increasing bioactive compounds

From a horticultural prospect, optimizing *T. officinale* cultivation for pharmaceutical purposes requires consideration of multiple factors that influence secondary metabolite biosynthesis. Environmental conditions, including light intensity, temperature, soil composition, and water availability, significantly impact the accumulation of bioactive compounds (Ianovici, 2016).

Research demonstrates that experimental conditions can trigger enhanced production of secondary metabolites, suggesting potential strategies for improving therapeutic potency through controlled cultivation practices (**Table 1**).

**Table 1:** Scientific studies on cultivation and elicitation techniques applied to *T. officinale*, aimed at enhancing the concentration of key bioactive compounds.

Cultivation technique/elicitor	Compounds increased	Main findings	Experimental notes	References
Salt stress (NaCl: 0–4 g/kg) in soil cultivation	Chlorogenic and isochlorogenic acids	Low salinity (1 g/kg) → ↑caffeoylquinic acids; High salt ↓caftaric and cichoric acids	Greenhouse conditions, HPLC + RT-PCR analysis	Zhu et al. (2022)
Hydroponic fertigation with iodine (KIO_3_) and bromine (KBr) at 10–50 μM	Chlorogenic acid, total phenols	Iodine 50 μM → ↑leaf phenols; Bromine 10 μM → ↑root phenols	Applied separately and in combination; LC–MS/MS analysis	Ledwożyw-Smoleń et al. (2025)
Hydroponics with pH-controlled nutrient solution (pH 4.0–7.0)	Total phenols, carotenoids, chlorophylls	pH 4.0 → ↑phenolics and pigments, ↓nitrates	Floating hydroponic system, greenhouse conditions	Alexopoulos et al. (2021)
Root-derived suspension culture + abiotic elicitors: methyl jasmonate (0.2 mM), β-cyclodextrin (25 mM)	Taraxasterol, taraxerol	β-cyclodextrin at 25 mM → TX 0.036%, TA 0.023%	Callus grown in MS with 2,4-D, BA, IAA; RP-HPLC analysis	Sharma and Zafar (2016)
Suspension culture from callus (MS medium + NAA + BAP); varied callus age, inoculum size, sucrose	α-Amyrin, lupeol	Optimal yield: 0.04 mg/g (α-amyrin), 0.03 mg/g (lupeol) at 8 weeks	Best conditions: 6-week-old callus, 4% inoculum, 1% sucrose	Martínez et al. (2023)

Zhu et al. (2022) assessed *T. officinale* grown under controlled conditions (12/12 h light, 25/20 ± 2°C, 125 µmol·m^-^²·s^-^¹) exposed to soil NaCl (0.5, 1, 2, 4 g·kg^-^¹) for 24 days. Growth was unaffected at ≤1 g·kg^-^¹, whereas 4 g·kg^-^¹ reduced fresh and dry weight to ~28% and ~42% of control, respectively. Caftaric and cichoric acids declined progressively with NaCl (to 29.5% and 52.6% of control at 4 g·kg^-^¹), while chlorogenic acid and isochlorogenic A peaked under mild stress (isochlorogenic A = 1.14× at 0.5 g·kg^-^¹ and 1.44× at 1 g·kg^-^¹). ToC3′H expression was upregulated at ≤1 g·kg^-^¹ (ToPAL peaked ~8.6× at 0.5 g·kg^-^¹), linking gene regulation to increased caffeoylquinic acids. Oxidative markers (MDA, REL) and proline rose at ≥2 g·kg^-^¹. The authors conclude mild salt stress (≤1 g·kg^-^¹) can be considered as a feasible abiotic elicitation to enhance caffeoylquinic compounds in *T. officinale*.

Ledwożyw-Smoleń et al. (2025) conducted a two-year pot experiment testing KIO_3_ and KBr (10 µM and 50 µM, alone and combined) on *T. officinale* grown in a tunnel. Results show a differential modulation of phenolic compounds by KIO_3_ and KBr fertigation across dandelion organs. In leaves, the highest iodine treatment (50 µM I) produced the largest increases in chlorogenic acid and total phenolics relative to control and other treatments, with chlorogenic acid reaching approximately ~399.5 mg·kg^-^¹ DW and concomitant rises in antioxidant capacity. By contrast, roots showed a significant increase in chlorogenic acid and total phenolics only following the low-dose bromine treatment (10 µM Br); most I and I+Br regimes did not consistently elevate root phenolics. These results indicate that iodine application is more effective at enhancing foliar phenolics (potentially improving the nutraceutical value of aerial tissues), whereas low-dose bromine can selectively boost root phenolics; however, the marked accumulation of Br (and high Br:I ratios) cautions for careful monitoring when designing biofortification strategies.

Low-pH nutrient solutions acted as a eustress able to enhance the phytochemical quality of *T. officinale* grown in floating hydroponics (Alexopoulos et al., 2021). The trial comparing pH 4.0, 5.5 and 7.0, plants at pH 5.5 showed the best overall biomass, but pH 4.0 significantly increased leaf phenolics content (≈735–758 mg GAE·kg^-^¹ FW at pH 4.0 across harvests). Although pH 4.0 reduced rosette diameter and root fresh weight modestly, it did not prohibit growth, suggesting a trade-off between yield and bioactive enrichment. The authors conclude that manipulating nutrient solution pH (notably mild acidification to pH 4.0) could be a practical cultivation strategy to boost phenolic and antioxidant quality in hydroponic dandelion.

Sharma and Zafar (2016) demonstrated that elicitation of *T. officinale* root callus suspension cultures with methyl jasmonate (MJ) and β-cyclodextrin (CD) markedly increased taraxasterol (TX) and taraxerol (TA) levels compared with natural root extracts. MJ (0.2 mM) raised TX from 0.0299% to 0.032% w/w (+7.0%) and TA from 0.0169% to 0.018% w/w (+6.5%). CD (25 mM) proved more effective, elevating TX to 0.036% w/w (+20.4%) and TA to 0.023% w/w (+36.1%). Maximal accumulation was observed in 21-day cultures following 25 days of elicitation, indicating CD-mediated elicitation as a particularly promising strategy to enhance *in vitro* yields of TX and TA.

In another study (Martínez et al., 2023), cell suspension cultures of *T. officinale* derived from hypocotyl callus were optimized for α-amyrin and lupeol production under varying inoculum density (0.2–8% w/v), callus age (2–10 weeks), and sucrose concentration (1–5.5% w/v). The most favorable conditions were achieved using a 6-week-old callus and 1% sucrose, which resulted in 0.04 ± 0.02 mg g^-^¹ α-amyrin and 0.03 ± 0.01 mg g^-^¹ lupeol after eight weeks of culture. Both callus maturity and carbon source concentration significantly influenced cell biomass, viability, and triterpene accumulation. These parameters were identified as critical determinants for establishing reproducible suspension cultures and serve as a foundation for subsequent elicitation strategies aimed at further enhancing triterpenoid biosynthesis in *T. officinale*.

## 5 Prospects

Although several studies have addressed conventional and/or open-field cultivation techniques (Gorenjak et al., 2012; Bretzel et al., 2014; Maleci et al., 2014; Basso et al., 2024; Shtakal et al., 2024), the transition of *T. officinale* toward a pharmaceutical-grade crop requires integrated horticultural strategies that maximize bioactive compound production while ensuring environmental and economic sustainability. Therefore, future research should prioritize the development of controlled environment agriculture systems, particularly hydroponic cultivation integrated with tailored LED lighting, as these technologies offer unprecedented opportunities for standardizing secondary metabolite profiles critical for leukemia and lymphoma therapy (Sankhuan et al., 2022; Inthima and Supaibulwatana, 2024).

### 5.1 Hydroponic system optimization and LED spectrum manipulation

Emerging evidence demonstrates that light quality profoundly influences phytochemical biosynthesis in medicinal plants (Tsivelika et al., 2025). For *T. officinale*, systematic investigation of LED spectral recipes—particularly red:blue:far-red ratios—across developmental stages could represent a high-priority research direction. Studies on related Asteraceae species show that specific wavelength combinations can double bioactive compound concentrations while maintaining economic biomass production (Sankhuan et al., 2022). Future work should establish compound-specific response curves for taraxasterol, sesquiterpenoid lactones, chlorogenic acid, and chicoric acid under various LED spectra, enabling precision cultivation protocols that maximize anticancer metabolite accumulation.

The integration of hydroponic nutrient management with LED optimization presents synergistic opportunities. Research has demonstrated that electrical conductivity manipulation (2.0-4.0 dS m^-^¹) enhances phenolic and terpenoid production in medicinal plants (Kim et al., 2018). Interestingly, the nutrient film technique has enabled the use of moderately saline water for vegetable production without compromising yield or quality, highlighting the potential of utilizing brackish water within a framework of sustainable agriculture (Palmitessa et al., 2024). For *T. officinale*, the mild acidification strategy (pH 4.0) combined with iodine biofortification (50 μM KIO_3_) shown to increase foliar phenolics could be integrated with blue-enriched LED spectra to further amplify chlorogenic and chicoric acid production—both compounds demonstrating significant anticancer activity (Fulga et al., 2023; Tsivelika et al., 2025). Conversely, root-targeted triterpenoid production might benefit from red-enriched spectra (Xie et al., 2021; Soun-udom et al., 2024) combined with low-dose bromine fertigation (Ledwożyw-Smoleń et al., 2025) and controlled EC stress (Shirazi et al., 2019).

### 5.2 Elicitation strategies and biotechnology applications

Strategic elicitation represents a powerful approach for enhancing pharmaceutical compound production. The demonstrated efficacy of β-cyclodextrin (25 mM) in elevating taraxasterol and taraxerol (Sharma and Zafar, 2016) suggests that integrated elicitation protocols—combining chemical elicitors with abiotic stress and optimized LED spectra—could achieve multiplicative enhancement effects (Kim et al., 2018). Future research should investigate temporal elicitation strategies, applying stress stimuli during final growth stages to maximize bioactive accumulation without severely compromising biomass.

Adventitious root culture systems warrant expanded investigation for producing root-specific anticancer compounds. These cultures allow targeted production of taraxasterol, β-amyrin, and sesquiterpenoid lactones under highly controlled conditions, with potential for bioreactor scaling (Rahmat and Kang, 2019). Developing optimal medium compositions, growth regulator combinations, and elicitation protocols for *T. officinale* root cultures could establish commercially viable production systems ensuring year-round supply of standardized pharmaceutical material.

### 5.3 Germplasm development and vertical farming

Substantial phytochemical variation among *T. officinale* populations indicates significant genetic improvement potential. Future breeding programs should prioritize: (1) systematic germplasm characterization to identify elite chemotypes with enhanced anticancer compound profiles; (2) development of molecular markers for trait selection; (3) establishment of clonal propagation systems to maintain desirable characteristics. Plant factories with artificial lighting (PFAL) could offer optimal environments for producing pharmaceutical-grade T*. officinale*, providing complete environmental control, contamination elimination, and space-efficient vertical production (Guo et al., 2025).

### 5.4 Sustainability and economic viability

Achieving economic viability requires optimizing production for pharmaceutical value rather than biomass yield. Energy-efficient LED systems, renewable energy integration, and recycled hydroponic solutions can minimize environmental footprint (Giménez et al., 2024) while maintaining profitability. Development of good agricultural and collection practices (GACP) specific to pharmaceutical *T. officinale* will ensure quality consistency, regulatory compliance, and market acceptance.

### 5.5 Translational pathway

The ultimate success of horticultural innovations depends on clinical validation. Well-designed trials using standardized, CEA-produced *T. officinale* extracts are essential for establishing safety and efficacy in leukemia and lymphoma patients. Collaborative efforts integrating horticulturists, analytical chemists, pharmacologists, and clinicians will be crucial for translating cultivation advances into therapeutic reality, potentially delivering safe, effective, and sustainable plant-based cancer treatments.

## 6 Conclusions

*T. officinale* contains promising bioactive metabolites with reproducible preclinical activity against leukemia and lymphoma. To translate these findings, priority must be given to standardizing plant material and extracts, performing pharmacokinetic/toxicology studies, and assessing drug–herb interactions. Horticultural strategies (controlled cultivation, elicitation, spectral and nutrient management) can reliably enrich target compounds and supply material for translational work.
